# Metagenomic analysis of the microbiota in the highly compartmented hindguts of six wood- or soil-feeding higher termites

**DOI:** 10.1186/s40168-015-0118-1

**Published:** 2015-11-26

**Authors:** Karen Rossmassler, Carsten Dietrich, Claire Thompson, Aram Mikaelyan, James O. Nonoh, Rudolf H. Scheffrahn, David Sillam-Dussès, Andreas Brune

**Affiliations:** Department of Biogeochemistry, Max Planck Institute for Terrestrial Microbiology, Marburg, Germany; Present address: Department of Civil and Environmental Engineering, Colorado State University, Fort Collins, CO USA; LOEWE Center for Synthetic Microbiology (Synmikro), Philipps-Universität Marburg, Marburg, Germany; Fort Lauderdale Research and Education Center, University of Florida, Davie, FL USA; Laboratory of Experimental and Comparative Ethology, University Paris 13, EA4443 Sorbonne Paris Cité, Villetaneuse, France; Institute of Research for Development—Sorbonne Universités, Institute of Ecology and Environmental Sciences, U242 Bondy, France

**Keywords:** Metagenomics, Termites, Hindgut, Functional potential, Gut microbiota, Community structure

## Abstract

**Background:**

Termites are important contributors to carbon and nitrogen cycling in tropical ecosystems. Higher termites digest lignocellulose in various stages of humification with the help of an entirely prokaryotic microbiota housed in their compartmented intestinal tract. Previous studies revealed fundamental differences in community structure between compartments, but the functional roles of individual lineages in symbiotic digestion are mostly unknown.

**Results:**

Here, we conducted a highly resolved analysis of the gut microbiota in six species of higher termites that feed on plant material at different levels of humification. Combining amplicon sequencing and metagenomics, we assessed similarities in community structure and functional potential between the major hindgut compartments (P1, P3, and P4). Cluster analysis of the relative abundances of orthologous gene clusters (COGs) revealed high similarities among wood- and litter-feeding termites and strong differences to humivorous species. However, abundance estimates of bacterial phyla based on 16S rRNA genes greatly differed from those based on protein-coding genes.

**Conclusion:**

Community structure and functional potential of the microbiota in individual gut compartments are clearly driven by the digestive strategy of the host. The metagenomics libraries obtained in this study provide the basis for future studies that elucidate the fundamental differences in the symbiont-mediated breakdown of lignocellulose and humus by termites of different feeding groups. The high proportion of uncultured bacterial lineages in all samples calls for a reference-independent approach for the correct taxonomic assignment of protein-coding genes.

**Electronic supplementary material:**

The online version of this article (doi:10.1186/s40168-015-0118-1) contains supplementary material, which is available to authorized users.

## Background

Termites are important contributors to carbon and nitrogen cycling in tropical ecosystems. Their ability to degrade lignocellulose is based on a partnership with a diverse community of microbial symbionts harbored in their intestinal tracts [1, 2].

While the evolutionarily lower termites have relatively simple guts and digest wood with the help of cellulolytic protists, the hindguts of higher termites are more strongly compartmented and contain exclusively prokaryotic microbial communities [1–3]. The individual gut compartments feature steep axial and radial gradients in physical parameters, such as pH, redox potential, and oxygen and hydrogen partial pressure [4–6], and microbial community structures along the intestinal tract strikingly differ between compartments [6–8].

Several metagenomic studies have assessed the functional potential of the gut microbiota in a few higher termites (including wood-feeding, dung-feeding, and fungus-cultivating species) but were usually restricted to the luminal contents [9–11]. These analyses revealed intriguing differences in the functional role of the microbiota in symbiotic digestion of lignocellulose, but the functional potential of the microbiota in individual compartments and differences to higher termites feeding on humus or soil are still entirely in the dark.

## Methods

### Sampling

The wood feeder *Microcerotermes parvus*, the litter feeder *Cornitermes* sp., the humus feeders *Termes hospes* and *Neocapritermes taracua*, and the soil feeder *Cubitermes ugandensis* were collected in the field; the wood feeder *Nasutitermes corniger* was from a laboratory colony. Species were initially identified according to morphology, and the identity was corroborated by mitochondrial genome analysis [12]. Guts of 30–50 worker termites were dissected into individual compartments, and DNA was extracted from pooled sections using a bead-beating protocol. Detailed information on the origin of the termites and sample processing can be found in the Additional file 1: Supplementary methods.

### Amplicon sequencing and analysis

The bacterial diversity in the different gut compartments was analyzed by paired-end sequencing of the 16S rRNA genes [13] on an Illumina MiSeq platform using the same DNA preparations as for metagenomic sequencing and bacteria-specific primers (V4 region). Amplicon sequencing yielded between 44,000 and 138,000 quality-filtered and trimmed sequences (iTags) per sample (see Table 1 for accession numbers). Reads were classified to the genus level using a curated reference database for the classification of dictyopteran gut microbiota (DictDb) [14].

**Table 1:** Summary of sample information and metagenomic library characteristics

Termite species strain mitogenome^a^ (Diet)	Gut section	Sample size (Gbp)	Assem. fraction (%)	Assy. size (Mbp)^b^	Contigs >50 kbp	Contigs >100 kbp	Longest contig (kbp)	IMGobject ID^c^	SRA acc. no.^d^
*Nasutitermes corniger* Nc150 KP091691 (wood)	C	4.5	59.9	338	0	0	10	1542	4604
	M	2.9	45.4	129	11	1	113	1466	4605
	P1	44.7	99.3	1425	10	0	84	2238	4606
	P3	46.8	94.7	635	44	5	296	2119	4607
	P4	42.9	94.7	1644	22	6	220	2308	4608
	P5	5.1	61.2	361	0	0	21	1343	4609
*Microcerotermes parvus* Mp193 KP091690 (wood)	P1	47.6	97.7	1476	0	0	39	2507	4601
	P3	43.2	95.1	712	9	0	71	2449	4602
	P4	48.0	97.1	1490	42	2	112	2509	4603
*Cornitermes* sp. Co191 KP091688 (litter)	P1	45.8	93.5	1534	267	42	249	2552	4592
	P3	45.9	91.5	1316	284	37	291	2450	4593
	P4	35.9	83.4	1303	8	2	275	2834	4594
*Termes hospes* Th196 KP091693 (humus)	P1	48.7	98.5	1511	12	0	85	2508	4613
	P3	34.2	82.2	1212	51	9	177	2469	4614
	P4	40.1	90.6	1800	50	3	110	2462	4615
*Neocapritermes taracua* Nt197 KP091692 (humus)	P1	43.9	91.6	1530	23	5	177	2501	4610
	P3	28.3	67.5	886	88	12	628	2505	4611
	P4	39.1	88.0	1537	42	14	232	2504	4612
*Cubitermes ugandensis* Cu122 KP091689 (soil)	C	3.2	40.5	111	0	0	12	1474	4595
	M	3.0	43.0	108	0	0	10	1468	4596
	P1	68.4	84.7	2060	31	2	176	2185	4597
	P3	32.0	71.2	1122	49	9	227	2127	4598
	P4	31.8	79.8	1295	15	2	107	2125	4599
	P5	37.6	96.8	1992	27	0	90	2175	4600

*C* crop (foregut), *M* midgut, *P1–P5* proctodeal compartments (hindgut)

^a^Accession numbers of mitochondrial genome sequence reconstructed from the metagenomes [12]

^b^Assembly size after dereplication

^c^IMG taxon object ID 330000*xxxx*

^d^NCBI sequence read archive accession number SAMN0334*xxxx*

### Metagenomic sequencing and analysis

Metagenomic libraries were prepared, sequenced, quality controlled, and assembled at the Joint Genome Institute (Walnut Creek, CA, USA). DNA was sequenced on an Illumina HiSeq 2000 (Illumina Inc., San Diego, CA). Quality-controlled reads were assembled and uploaded to the Integrated Microbial Genomes (IMG/M ER) database (https://img.jgi.doe.gov/cgi-bin/mer/main.cgi) for gene identification and annotation by applying the standard operation procedure of IMG [15]. The metagenomes are publicly available on the IMG/M ER website (see Table 1 for accession numbers). Gene functions of protein-coding genes were identified, and genes were taxonomically assigned using BLASTp (top hit) and RPS-BLAST against the COG database.

## Quality assurance

In addition to using standard precautions, we verified the reproducibility of the iTag data sets by comparing them to previously published data sets for the same termite species (or genus). We also conducted independent analyses of community structure in the same samples using libraries obtained with a different primer set (unpublished results). The absence of noteworthy differences also assured that our data sets were not contaminated.

## Initial findings

**Fig. 1:**
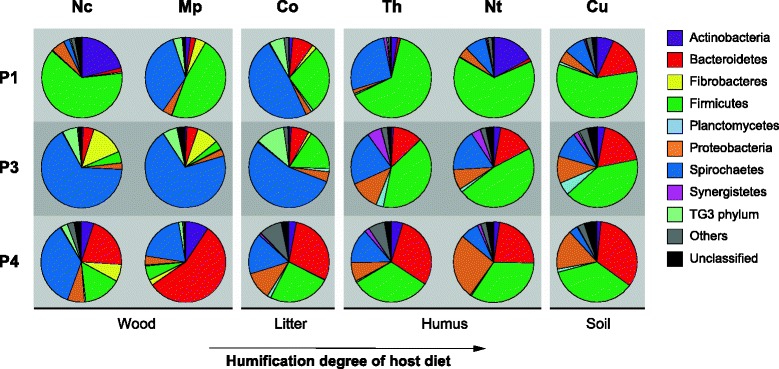
Bacterial community structure in the major hindgut compartments (P1, P3, and P4) of six termite species. The relative abundance of major bacterial phyla in the iTag analysis is shown; detailed classification down to the genus level is shown in Additional file 2: Table S1. Termite host abbreviations: *Nc Nasutitermes corniger*, *Mp Microcerotermes parvus*, *Co Cornitermes* sp., *Th Termes hospes*, *Nt Neocapritermes taracua*, *Cu Cubitermes ugandensis*

We analyzed amplicon libraries and metagenomic libraries obtained for six species of higher termites to compare the structure and functional potential of the intestinal microbiota in the major gut compartments. iTag sequencing analysis revealed strong differences in bacterial community structure already at the phylum level, both between the individual gut compartments of each termite and among the homologous gut compartments of termites with different feeding strategies (Fig. 1). Spirochaetes represented the majority of bacteria in the P3 compartment of wood and litter feeders but comprised only a minor proportion in the humus and soil feeders, which is in agreement with previous reports based on bacterial clone libraries obtained from total guts of congeneric species [8, 16, 17]. The presence of Fibrobacteres and the TG3 phylum exclusively in the gut microbiota of wood and litter feeders matches previous observations [17, 18] and the characteristic association of these lineages with wood fibers [19].

The bacterial community of the P1 compartment of most termite species was dominated by Firmicutes, which is in agreement with previous reports on the microbiota of this sometimes highly alkaline hindgut compartment [6–8]; the high proportions of Spirochaetes and Actinobacteria in certain termite species are exceptional but not unprecedented [6, 8]. The bacterial communities in the P4 were generally more diverse than in the other compartments and displayed an increasing abundance of Bacteroidetes, which matches previous observations with *Nasutitermes* and *Cubitermes* species [6, 7]. The detailed classification results for all taxonomic ranks down to the genus level are shown in Additional file 2: Table S1.

Metagenomic sequencing of the major hindgut compartments (P1, P3, and P4) of the six termite species yielded an average library size of 42 Gbp (range, 30–70 Gbp), with 90 % of the bases (range, 68–99 %) in the assembled fraction (Table 1). The large number of bacterial contigs longer than 100 kbp and the strong size reduction of the assemblies to 1.4 Gbp (range, 0.6–2.1 Gbp) after dereplication indicate a relatively low diversity of the respective communities. In a pilot experiment with *N. corniger* and *Cubitermes ugandensis*, we also obtained smaller libraries (3–5 Gbp) for the crop (foregut), midgut, and P5 compartments, with only 50 % of the bases in the assembled fraction. Because assembly sizes after dereplication were about tenfold smaller (0.1–0.4 Gbp) (Table 1), these datasets were not included in the following analyses.

**Fig. 2:**
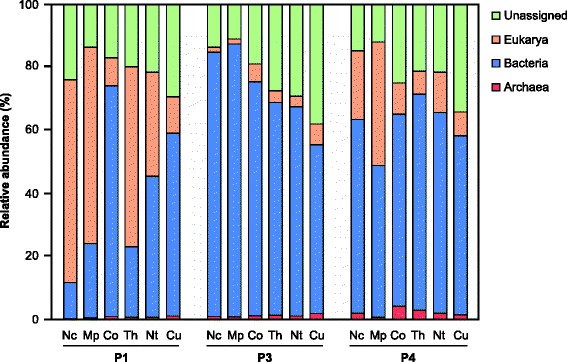
Assignment of protein-coding genes in the metagenomic libraries to the three top-level domains. Taxonomic assignment is based on BLASTp analysis (top hit >30 % identity). The abundance of a gene in a library was estimated using the length and read depth of the gene in the respective assembly (read depth of 1 for unassembled reads). Termite host abbreviations: *Nc Nasutitermes corniger*, *Mp Microcerotermes parvus*, *Co Cornitermes* sp., *Th Termes hospes*, *Nt Neocapritermes taracua*, *Cu Cubitermes ugandensis*

A BLASTp analysis of the metagenomes allowed assignment of the majority of the protein-coding genes to the three top-level domains; only 10–38 % of the gene copies remained unclassified (Fig. 2). In most libraries, the majority of genes were of bacterial origin. Archaeal genes represented only a small fraction of the gene copies in all libraries, with highest proportions (up to 4 %) in the P4 compartment, which is in agreement with the low abundance of archaeal rRNA in termite hindguts [20]. However, in most P1 compartments, bacterial genes were outnumbered by genes assigned to eukaryotes. Notable exceptions are *Cornitermes* sp. and *Cubitermes ugandensis*, where the P1 is almost as large as the P3 [7, 21]. This agrees with our expectation that the proportion of host DNA will be larger in smaller compartments (resulting in a higher surface-to-volume ratio and hence relatively more host tissue) and the observation that the density of the gut microbiota is generally lower in the P1 than in other compartments [6, 7]. Nevertheless, the remaining information on the bacterial and archaeal microbiota is sufficient to draw conclusions about symbiont-mediated functions in each gut compartment.

**Fig. 3:**
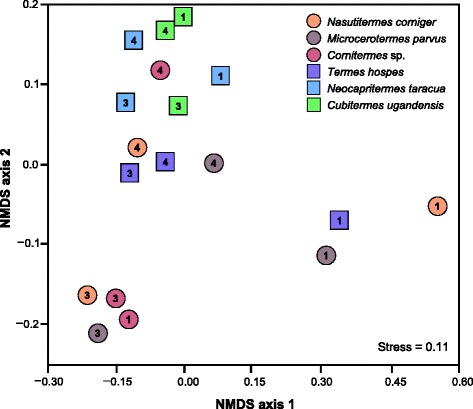
Similarity of the functional potential of the microbiota in different gut compartments. The analysis is based on non-metric multidimensional scaling (NMDS) of Bray-Curtis similarities using the relative abundances of genes in different functional categories (COGs), weighted by gene length and read depth in the respective assembly (see Additional file 2: Table S2). The shape of the data points differentiates wood and litter feeders (*circle*) from humus and soil feeders (*square*); *numbers* indicate gut compartments P1, P3, and P4

The differences in bacterial community structure among the gut compartments were reflected in the relative abundances of COG functional categories in the respective libraries, which indicated that the functional potential of the bacterial gut microbiota differs between feeding groups (Fig. 3). The tight clustering of the P3 compartments of wood- and litter-feeding termites, with the inclusion of the P1 compartment from the litter-feeding termite, indicates that patterns in functional potential of the gut microbiota are correlated with the feeding strategy of the host. In addition, P1 from wood-feeding termites, as well as P1 from the humus-feeding *T. hospes* clustered separately from other gut compartments, which indicates similarities between homologous gut compartments regardless of feeding strategy. Detailed results of the COG analysis are shown in Additional file 2: Table S2.

A comparison of the bacterial community structure determined by iTag analysis with the phylogenetic classification of protein-coding genes in the metagenomes revealed large discrepancies already at the phylum level (Additional file 2: Table S1). While Fibrobacteres and the TG3 phylum were highly abundant in bacterial communities of wood- and litter-feeding termites, they were strongly underrepresented (Fibrobacteres) or undetected (TG3 phylum) in the taxonomic assignments of the protein-coding genes (exemplified in Additional file 3: Figure S1). This discrepancy is explained by the lack of appropriate reference genomes in public databases. The only sequenced genome from Fibrobacteres, the rumen isolate *Fibrobacteres succinogenes*, is only distantly related to Fibrobacteres detected in this study [22], and the draft genome of *Chitinivibrio alkaliphilus*, the first isolate of the TG3 phylum [23], was not included in public databases at the time of analysis. The high abundance of genes assigned to Proteobacteria, which contrasts strongly with their low proportion in the iTag datasets, is also likely caused by the bias introduced by incorrect assignment due to the lack of reference genomes.

## Future directions

The results of this preliminary analysis show that microbial structure and function are correlated with both the digestive strategy of the host and corresponding microhabitats. The large metagenomic datasets will allow an in-depth analysis of the microbial functions in the homologous gut compartments and a comparison between hosts with diverging digestive strategies. Of particular interest will be the gene functions related to the digestion of lignocellulose and the putative peptidic substrates in the diet of the humivorous host [1]. To overcome the bias in the taxonomic assignment of the genes, we are currently using a reference-independent approach to reconstruct population genomes for the major lineages of uncultivated symbionts.

## Availability of supporting data

Metagenomes are available at the Integrated Microbial Genome database (http://img.jgi.doe.gov). 16S rRNA gene sequences (iTags) have been deposited in the NCBI Sequence Read Archive (http://ncbi.nlm.nih.gov/sra). Taxon object IDs and accession numbers for each sample are listed in Table 1.

## Additional files

Additional file 1:
**Supplemental methods.** Detailed description of metagenome and amplicon sample collection and processing, sequencing, and analysis. (DOCX 39 kb) Additional file 2:
**Supplemental tables.** Microsoft Excel file containing three sheets (Tables S1–3) that contain the abundance and taxonomic information of iTag, COG, and protein-coding genes. (XLSX 3094 kb) Additional file 3: Figure S1. Supplemental figures. Data visualization supporting the statements of the main text. (DOCX 61 kb)
