# Supplementary material for: Metagenomic analysis of the microbiota in the highly compartmented hindguts of six wood- or soil-feeding higher termites
Source: Microbiome. 2015 Nov 26;3:56. doi: 10.1186/s40168-015-0118-1 (PMC4660790; doi:10.1186/s40168-015-0118-1)
Supplement: Additional file 1: — Supplemental methods. Detailed description of metagenome and amplicon sample collection and processing, sequencing, and analysis. (DOCX 39 kb) [file 40168_2015_118_MOESM1_ESM.docx]

# ADDITIONAL FILE 1 - Supplementary methods

Metagenomic analysis of the microbiota in the highly compartmented hindguts of six wood- and soil-feeding higher termites

Karen Rossmassler, Carsten Dietrich, Claire Thompson, Aram Mikaelyan, James Nonoh, Rudolf H. Scheffrahn, David Sillam-Dussès and Andreas Brune

## Sample collection and preparation

*Cornitermes* sp. (Co191), and *Neocapritermes taracua* (Nt197) were collected near Petit Saut dam, French Guiana. *Termes hospes* (Th196) and *Microcerotermes parvus* (Mp193) were collected near Pointe-Noire, Democratic Republic of the Congo. *Cubitermes* *ugandensis* (Cu122) were collected on Lhiranda Hill in Kakamega Forest, Kenya. *Nasutitermes corniger* (Nc150) were obtained from a laboratory-maintained colony (University of Florida). Species identity was established by morphology and by reconstruction of the complete mitochondrial genome sequences [1].

For each termite species, the guts of 30–50 workers were dissected with fine-tipped forceps into individual compartments (C, crop; M, midgut; P1–P5, proctodeal compartments 1–5). Gut sections were pooled in 2-ml polypropylene tubes containing 100 µL phosphate-buffered saline (pH 7.2) and stored at –20 °C until extraction [2]. DNA was extracted using the NucleoSpin Soil kit with SL2 lysis buffer and SX buffer (Macherey-Nagel). DNA was quantified with Quant-IT dsDNA Assay on a Qubit fluorometer (Life Technologies), and purity was checked with a Nanodrop 1000 spectrophotometer (Peqlab).

## Amplicon sequencing and analysis

The bacterial diversity in the different gut compartments was analyzed by Illumina-based analysis [3]. Using the same DNA preparations as for metagenomic analysis, the V4 region of the 16S rRNA genes was amplified with the forward primer 515F (5’-GTGCCAGCMGCCGCGGTAA-3’) and the reverse primer 806R (5’-GGACTACHVGGGTWTCTAAT-3’) as described in [4]. Amplicon sequencing on an Illumina MiSeq platform yielded between 44,000 and 138,000 quality-filtered and trimmed sequences (iTags) per sample (SRA acc. nos. in Table 1 in the corresponding article). Sequences were then dereplicated and aligned using the aligner implemented in the *mothur* software v1.33 [5]. Aligned sequences were assigned to taxonomic groups using the naïve Bayesian classifier implemented in *mothur* at a confidence threshold of 80% in combination with a manually curated reference database DictDb v.3.5 of bacterial lineages specific to termite guts [6].

## Metagenomic sequencing and analysis

100 ng of DNA was sheared to 270-bp fragments using an E210 ultrasonicator (Covaris) and size-selected using SPRI magnetic beads (Beckman Coulter). The fragments were treated with end-repair, A-tailing, and ligation of Illumina-compatible adapters (IDT, Inc) using the KAPA-Illumina library creation kit (KAPA Biosystems).

Libraries were quantified using KAPA Biosystem’s next-generation sequencing library qPCR kit and run on a Roche LightCycler 480 real-time PCR instrument. The quantified libraries were then prepared for sequencing on the Illumina HiSeq sequencing platform with a TruSeq paired-end cluster kit, v3-cBot-HS, and Illumina’s cBot instrument to generate clustered flow cells for sequencing. Sequencing of the flow cells was performed on the Illumina HiSeq 2000 sequencer using TruSeq SBS sequencing kits, v3-cBot-HS, following a 2x150 indexed-run recipe.

Quality-controlled reads were assembled using SOAPdenovo v1.05 [7] at a range of six kmers (85, 89, 93, 97, 101, 105) with the default settings. The six contig sets were dereplicated and sorted based on length. Contigs shorter than 1800 bp were assembled into longer contigs using Newbler (Life Technologies, Carlsbad, California, USA).

Protein-coding genes were identified from predicted open reading frames and assigned to phylogenetic bins using BLASTp (top hit, 30% identity cutoff). Gene functions were predicted also using RPS-BLAST against the COG database [8]. If no taxonomic information was available, genes were labeled as “unassigned”. COG annotations of ORFs identified to be prokaryotic were assigned to functional categories, and their relative abundances in each metagenome were visualized using non-metric multidimensional scaling (NMDS) implemented in PAST v.3.0 [9].

# References

1. Dietrich C, Brune A: **The complete mitogenomes of six higher termite species reconstructed from metagenomic datasets (*Cornitermes* sp., *Cubitermes ugandensis, Microcerotermes parvus, Nasutitermes corniger, Neocapritermes taracua*, and *Termes hospes*)**. *Mitochondrial DNA* 2014, early online (http://dx.doi.org/10.3109/19401736.2014.987257).

2. Köhler T, Dietrich C, Scheffrahn RH, Brune A: **High-resolution analysis of gut environment and bacterial microbiota reveals functional compartmentation of the gut in wood-feeding higher termites (Nasutitermes spp.)**. *Appl Environ Microbiol* 2012, **78**:4691–4701.

3. Degnan PH, Ochman H: **Illumina-based analysis of microbial community diversity**. *ISME J* 2012, **6**:183–194.

4. Caporaso JG, Lauber CL, Walters WA, Berg-Lyons D, Lozupone CA, Turnbaugh PJ, Fierer N, Knight R: **Global patterns of 16S rRNA diversity at a depth of millions of sequences per sample**. *Proc Natl Acad Sci* 2011, **108**:4516–4522.

5. Schloss PD, Westcott SL, Ryabin T, Hall JR, Hartmann M, Hollister EB, Lesniewski RA, Oakley BB, Parks DH, Robinson CJ, Sahl JW, Stres B, Thallinger GG, Van Horn DJ, Weber CF: **Introducing mothur: Open-source, platform-independent, community-supported software for describing and comparing microbial communities**. *Appl Environ Microbiol* 2009, **75**:7537–7541.

6. Mikaelyan A, Köhler T, Lampert N, Rohland J, Boga H, Meuser K, Brune A: **Classifying the bacterial gut microbiota of termites and cockroaches: a curated phylogenetic reference database (DictDb)**. *Syst Appl Microbiol*, in revision.

7. Li R, Zhu H, Ruan J, Qian W, Fang X, Shi Z, Li Y, Li S, Shan G, Kristiansen K, Li S, Yang H, Wang J, Wang J: **De novo assembly of human genomes with massively parallel short read sequencing**. *Genome Res* 2010, **20**:265–272.

8. Tatusov RL, Fedorova ND, Jackson JD, Jacobs AR, Kiryutin B, Koonin E V, Krylov DM, Mazumder R, Mekhedov SL, Nikolskaya AN, Rao BS, Smirnov S, Sverdlov A V, Vasudevan S, Wolf YI, Yin JJ, Natale DA: **The COG database: an updated version includes eukaryotes.** *BMC Bioinformatics* 2003, **4**:41.

9. Hammer Ø, Harper DAT, Ryan PD: **PAST: Paleontological Statistics Software Package for Education and Data Analysis. v.2.17**. *Palaeontol Electron* 2001, **4**:1–92.
