# Supplementary material for: Metagenomic analysis of the microbiota in the highly compartmented hindguts of six wood- or soil-feeding higher termites
Source: Microbiome. 2015 Nov 26;3:56. doi: 10.1186/s40168-015-0118-1 (PMC4660790; doi:10.1186/s40168-015-0118-1)
Supplement: Additional file 3: Figure S1. — Supplemental figures. Data visualization supporting the statements of the main text. (DOCX 61 kb) [file 40168_2015_118_MOESM3_ESM.docx]

# ADDITIONAL FILE 3 - Supplementary figure

Metagenomic analysis of the microbiota in the highly compartmented hindguts of six wood- and soil-feeding higher termites

Karen Rossmassler, Carsten Dietrich, Claire Thompson, Aram Mikaelyan, James Nonoh, Rudolf H. Scheffrahn, David Sillam-Dussès and Andreas Brune

**Figure S1. Relative abundances of the major bacterial phyla in iTag libraries and metagenomes.** The relative abundances of the protein-coding genes were estimated using length and read depth of the gene in the respective assembly (read depth of 1 for unassembled reads). Detailed results for individual species and gut compartments are shown in Additional File 3 (Table S3).
